# Fear of COVID-19 and COVID-19 Stress and Association with Sociodemographic and Psychological Process Factors in Cases under Surveillance in a Frontline Worker Population in Borneo

**DOI:** 10.3390/ijerph18137210

**Published:** 2021-07-05

**Authors:** Nicholas Tze Ping Pang, Gracyvinea Nold Imon, Elisa Johoniki, Mohd Amiruddin Mohd Kassim, Azizan Omar, Syed Sharizman Syed Abdul Rahim, Firdaus Hayati, Mohammad Saffree Jeffree, Jun Rong Ng

**Affiliations:** Faculty of Medicine and Health Sciences, Universiti Malaysia Sabah, Kota Kinabalu 88400, Sabah, Malaysia; nicholas@ums.edu.my (N.T.P.P.); evniagrace96@yahoo.com (G.N.I.); elisavitalis@yahoo.com (E.J.); azizan.omar@ums.edu.my (A.O.); syedsharizman@ums.edu.my (S.S.S.A.R.); m_firdaus@ums.edu.my (F.H.); saffree@ums.edu.my (M.S.J.); ryanng.95.jr@gmail.com (J.R.N.)

**Keywords:** fear of COVID-19, COVID-19 stress, psychological flexibility, mindfulness, persons under investigation

## Abstract

COVID-19 stress and fear of COVID-19 is an increasingly researched construct in the general population. However, its prevalence and association with sociodemographic factors and psychological process variables has not been explored in frontline workers under surveillance in a Bornean population. This study was a cross-sectional study using a sociodemographic questionnaire incorporating two specific epidemiological risk variables, namely specific questions about COVID-19 surveillance status (persons under investigation (PUI), persons under surveillance (PUS), and positive cases) and the nature of frontline worker status. Furthermore, five other instruments were used, with three measuring psychopathology (namely depression, anxiety and stress, fear of COVID-19, and stress due to COVID-19) and two psychological process variables (namely psychological flexibility and mindfulness). Kruskal–Wallis and Mann–Whitney tests were performed to assess if there were significant differences in psychopathology and psychological process variables between sociodemographic and epidemiological risk variables. Hierarchical multiple regression was further performed, with depression, anxiety, and stress as dependent variables. There were significant differences in the fear of COVID-19 between positive cases, PUI, and PUS. The fear of COVID-19 scores were higher in positive cases compared to in PUS and PUI groups. Upon hierarchical multiple regression, mindfulness and psychological flexibility were significant predictors of depression, anxiety, and stress after controlling for sociodemographic and epidemiological risk factors. This study demonstrates that exposure to COVID-19 as persons under investigation or surveillance significantly increases the fear of COVID-19, and brief psychological interventions that can positively influence mindfulness and psychological flexibility should be prioritized for these at-risk groups to prevent undue psychological morbidity in the long run.

## 1. Introduction

COVID-19 was first reported in China back in December 2019 [1] and was subsequently declared by the World Health Organization (WHO) as a pandemic on 11 March 2020 [2]. Echoing efforts by many countries worldwide, Malaysia implemented a movement control order (MCO) back in 18 March 2020 in light of increasing cases [3,4]. Apart from movement and social restrictions, extensive contact tracing and large-scale quarantine for positive cases and at-risk groups were performed. These aggressive measures reduced the reproduction number, R-naught (Rt), to less than one in May 2020, signifying a lower infectivity rate [5]. However, after three months of quiescence and relaxation of lockdowns, positive case figures spiked again after the Sabah state election in September 2020, resulting in a significantly more debilitating third wave of infections [6]. Two weeks prior the election, there was 9882 positive cases in Malaysia (as of 12 September 2020). However, 58,847 cases were reported as of 24 November 2020, signifying a 48,965 case increment in just two months [7].

The third wave resulted in unprecedented challenges to mental health, particularly in positive cases and at-risk groups. Over 37,000 phone calls were made to mental health helplines, illustrating growing mental health concerns nationally [8]. Worryingly, there were 266 suicides throughout the MCO, translating into one suicide case per day, with debt caused by job losses and family problems cited as the main factors [9]. A common theme was secondary consequences of the pandemic and social isolation as major lifestyle and economic disruptions re-ensued, associated with recurrent social standstill and economic decline [10]. Due to the reimplementation of movement control measures, social isolation and re-adaptation of new norms resulted in significantly elevated psychological distress due to depression and anxiety associated with childcare issues, food insecurity, reduced access to routine medical care, symptoms ascribed to COVID-19, and lack of daily structure [11]. In terms of educational attainment, due to reduced family income during the pandemic secondary to economic shutdowns [12], tertiary education was increasingly difficult to finance privately, contributing to increases in emotional distress among the student population [13].

In addition, a particular at-risk group for further psychological sequelae are COVID-19 patients and close contacts. This is related to fear of personal infection or infection of friends and family, as well as disruption in social and occupational functioning [14,15]. However, Malaysian research into such sequelae remains sparse. Similar Ecuadorian studies reveal no significant difference in the prevalence of emotional distress among suspected and confirmed COVID-19 cases. However, the distribution of severity showed that higher symptom endorsement was observed among the confirmed group [16]. A group of close contacts requiring particular attention is healthcare workers, who uniformly suffer elevated emotional distress during COVID-19 [17,18,19,20,21,22], resulting in high levels of depression, anxiety, and insomnia [22]. Moreover, there was a shortage of supply of protective equipment in the early pandemic, requiring healthcare workers to conserve usage of protective clothing, resulting in discomfort and fatigue [23,24]. Such fatigue and burnout were correlated with progressive increases in workload [23,25]. Similarly, themes of fear of spreading infection to family and loved ones also prevailed [18,21]. Hence, it is crucial that there are studies done to examine the unique psychological sequelae on persons with close contact and positive cases, especially in healthcare workers, and identify particular underlying psychological factors that may influence the development of psychopathology.

To the best of our knowledge, Malaysian research is scant in examining and contrasting psychological distress among confirmed and suspected cases (persons under surveillance (PUS) and persons under investigation (PUI)) of COVID-19, as well as exploring underlying psychological process variables. This is key, as mindfulness, psychological flexibility, and psychological mindedness (PM) have been determined as factors that can directly affect the levels of psychological distress [26,27,28,29]. Mindfulness can be defined as focusing one’s present moment thoughts, feelings, and sensations open-mindedly, without attempting to change the experience [30]. Meanwhile, psychological mindedness involves the ability of a person to recognize meanings behind words and actions, to appreciate emotional overtone and complexity, identify between past and present, and have insight into one’s intentions [31,32,33]. On the other hand, psychological flexibility is the ability to fully experience the present moment that includes one’s thoughts and feelings without struggling to control or change it, and the ability to either persist or change behavior in the given context that is consistent with one’s values and goals [34]. 

Globally, there are only limited studies exploring the psychological burden suffered by COVID-19 patients or their close contacts [35,36,37,38]. Hence, this study aims to explore the mental health burden and associated psychological constructs among primarily COVID-19 patients and close contacts in a university population in Malaysia; previous literature has examined it qualitatively but not quantitatively, or has not looked at the mental health aspect of matters [39,40]. We will look at the effect of sociodemographic variables, psychological mindedness, psychological flexibility, and fear of COVID-19 upon depression, anxiety, and stress in positive cases, persons under investigation (PUI) who have been in close contact with positive cases, and persons under surveillance (PUS) who are at risk but have no close contact with positive cases. 

## 2. Materials and Methods

Ethics approval was obtained from the Universiti Malaysia Sabah (UMS) medical research ethics committee.

All interactions were done online as social contact was not permitted. A Google form was utilized to collect data. Convenience sampling was used for this interview. The inclusion criteria were individuals above 18 years of age who were persons under investigation, persons under surveillance, and positive cases that were picked up during surveillance of the COVID-19 Command Centre in UMS, and who were willing to participate in the study and were able to read and converse fluently in Malay. The exclusion criteria were non-consent, and acute medical or psychiatric illness.

A sociodemographic questionnaire was used to assess certain variables as follow: their epidemiological status (categorized as PUI, PUS, positive case, or none of the above); their status as frontline workers (whether they were healthcare worker (HCW) dealing with COVID cases directly, HCW not dealing with COVID cases directly (e.g., psychiatry, surgery), HCW doing public health COVID work, non-medical frontliners (e.g., student affairs, security); age; gender; educational level; length of work in number of years; city currently residing during the COVID-19 pandemic; and marital status. Positive cases referred to individuals who tested positive for COVID-19 infection on a polymerase chain reaction (PCR) or rapid test kit (RTK) set, namely serologically. Persons under investigation (PUI) as defined here referred to persons who were close contacts to COVID-19 positive cases and hence had to undergo home quarantine for a period of two weeks. Persons under surveillance (PUS) referred to individuals who were in contact with PUI and had symptoms, but were not in close contact with positive cases, and hence might not require swabbing, but were designated to home quarantine for two weeks as well. 

Five validated instruments were used to collect data as follows.

### 2.1. Fear of COVID-19 Scale (FCV-19S)

The Fear of COVID-19 Scale [41] consists of seven items (e.g., “It makes me uncomfortable to think about coronavirus-19”). It is scored on a five-item Likert point response ranging from 1 (strongly disagree) to 5 (strongly agree), with possible scores range from 7 to 35. Higher scores indicate more severe fears of COVID-19 [35]. In this study, a validated Malay version (36) was administered which had very good internal consistency, with Cronbach’s alpha of 0.893 and McDonald’s omega of 0.894 [42]. 

### 2.2. 21-Item Depression Anxiety and Stress Scales (DASS-21)

The DASS-21 [43] measures the severity of depression, anxiety, and stress. It consists of 21 items that measures three different domains: depression (e.g., “I felt downhearted and blue”), anxiety (e.g., “I was worried about situations in which I might panic and make a fool of myself”), and stress (e.g., “I found it hard to calm down after something upset me”). Each item is scored on a four-point Likert scale ranging from 0 (did not apply to me at all over the last week) to 3 (applied to me very much or most of the time over the past week). Higher scores in each domain correlate with greater severity of emotional distress. In this study, the Malay version of the DASS-21 [44] was administered, which showed acceptable Cronbach’s alpha values of 0.84, 0.74, and 0.79, respectively, for depression, anxiety, and stress. In addition, it had good factor loading values for most items (0.39 to 0.73) [38].

### 2.3. Acceptance and Action Questionnaire (AAQ II) 

The AAQ II [45] is a widely used measure of experiential avoidance and psychological inflexibility. It was developed and revised from the original AAQ [46]. It is a unidimensional scale with seven items and is rated based on a seven-point Likert scale, ranging from 1 (never true) to 7 (always true). The possible score ranges from 7 to 49. Greater scores on AAQ II indicate higher levels of psychological inflexibility. The Malay version of AAQ II was used in this study, which had a Cronbach’s alpha of 0.91, excellent parallel reliability, and adequate concurrent validity [47]. 

### 2.4. Mindfulness, Attention, and Awareness Scale (MAAS) 

The Mindfulness, Attention, and Awareness Scale (MAAS) is used to assess awareness and attention in everyday life. It is a 15-item scale which measures the frequency of mindful states in a day-to-day life, using both general and situation-specific statements. A range of scores from 1 to 6 is given for each item. Totals ranging from 15 to 90, with higher score indicating greater mindfulness [48]. In this study, the Malay version of the MAAS (MMAAS) was administered. The internal consistency of MMAAS was good (Cronbach’s α = 0.851) and has satisfactory psychometric properties [49]. 

### 2.5. Coronavirus Stress Measure (CSM)

The Coronavirus Stress Measure is a five-item scale adopted from the Perceived Stress Scale, which has been established as a valid and reliable measurement tool assessing COVID-19 related stress. It demonstrated satisfactory internal consistency reliability estimate, with Cronbach’s α values ranging from 0.83 to 0.96 for the scale [50]. The construct validity of the CSM was also assessed using confirmatory factor analyses, establishing a unidimensional structure comprising five items. The scale also showed good evidence of convergent validity with theoretically similar constructs, such as anxiety and depression, and divergent validity with demographic factors such as age. The Malay validation of the CSM was performed in a Malaysian population in a recent study that is pending publication [51], with adequate psychometric properties including Cronbach’s alpha of 0.891, and demonstrated significant correlations with stress (r = 0.632, *p* < 0.001), anxiety (r = 0.590, *p* < 0.001), and depression (r = 0.579, *p* < 0.001) subscales of the DASS-21.

### 2.6. Balanced Index of Psychological Mindedness (BIPM)

The Balanced Index of Psychological Mindedness is used for objective measurement of psychological mindedness level. It is a 14-item scale, with two factor models, namely interest and insight [52]. It is rated based on a five-point Likert scale, ranging from 0 (not true) to 4 (very true). Positive statements are scored based on the Likert scale, while negative statements are inversely scored. The totals range from 0–28, with higher scores indicating higher level of psychological mindedness. The BIPM demonstrated good internal consistency with Cronbach’s α of 0.85 and 0.76 for interest and insight, respectively, with good test/retest reliability (r = 0.63 and 0.71, respectively). The Malay version of the BIPM also showed good psychometric properties; Cronbach’s alpha for the insight and interest subscales was 0.87 and 0.82, respectively [33].

### 2.7. Data Analysis

IBM SPSS version 26.0 was employed for all statistical analysis. Descriptive statistics were used, with skewness and kurtosis calculated to assess if normalcy criteria were met. Subsequently, depending on normalcy assessments, suitable statistical tests were performed to assess if there was any difference between groups for all psychological variables. Correlation coefficients were performed to assess for bivariate relationships between all psychological variables. In the subsequent stage, hierarchical multiple regression was performed with all sociodemographic variables imputed in the first stage and all psychological variables imputed in the second stage. R-squared changes were reported for each stage of multiple regression.

## 3. Results

### 3.1. Demographic

The skewness and kurtosis were <+/2 for all continuous psychological variables. The ages of the participants ranged from 19–60 years old, with a mean of 30 years and a standard deviation of 7.94 years. The majority (29.3%) of the participants were PUIs (53 individuals), followed by 52 participants who were not exposed to COVID-19 (28.7%) and 51 PUS (28.2%), as shown in Table 1. Only 17 positive cases within the university were able to be recruited, as there were only a small number of positive cases amongst the university population. The majority of the participants (103 individuals) were not frontliners (56.9%), while 53 individuals (25.9%) were healthcare worker frontliners and 25 individuals (13.8%) were non healthcare worker frontliners. The majority of participants were females with Bachelor’s degrees who had been working for four or more years, and who were mostly living in Kota Kinabalu. 

### 3.2. Bivariate Correlations

As per Table 2, there were correlations between fear of COVID-19 and psychological mindedness, depression, anxiety, as well as COVID-19 stress and mindfulness. However, fear of COVID-19 was not correlated with COVID-19 stress. 

Psychological mindedness was correlated with psychological flexibility, depression, anxiety, and stress, but was not correlated with mindfulness.

### 3.3. Bivariate Tests

The Kruskal–Wallis test showed significant differences for the fear of COVID-19 scale between positive cases, persons under investigation, and persons under surveillance. The fear of COVID-19 scores were higher in positive cases compared to the PUS and PUI groups, as shown below in Table 3 and Table 4. 

The Kruskal–Wallis tests further showed significant differences in fear of COVID-19, psychological flexibility, anxiety, and stress for marital status. Interestingly, based on Table 5 and Table 6, married people had higher fear but lower psychological flexibility, depression, anxiety, and stress compared to single people.

The Kruskal–Wallis tests in Table 7 and Table 8 also showed significant differences in the fear of COVID-19, depression scores, and stress scores for different education levels.

Mann–Whitney tests were performed and tabulated as in Table 9, which demonstrated significantly higher scores for females compared to males in various dimensions, namely depression, anxiety, stress, COVID-19 related stress, and psychological flexibility.

### 3.4. Hierarchical Multiple Regression

A two-step model was employed whereby sociodemographic variables were inputted at the first step. Subsequently, psychological variables were inputted at the second step. Depression, anxiety, and stress were used as the dependent variables.

When depression was used as the dependent variable (Table 10 and Table 11), age was significant at the first step, but after the addition of psychological variables at the second step, age ceased to be significant. COVID-19 related stress, mindfulness, fear of COVID-19, and psychological flexibility were all significant predictors of depression.

When anxiety was used as the dependent variable (Table 12, Table 13 and Table 14), age was significant at the first step, but after addition of psychological variables, it ceased to be significant. Mindfulness and psychological flexibility were all significant predictors of anxiety.

When stress was used as the dependent variable (Table 15, Table 16 and Table 17), both age and male gender were significant at the first step. However, after addition of psychological variables, age became not significant, but male gender remained significant. Mindfulness and psychological flexibility were all significant predictors of stress.

## 4. Discussion

These findings suggest that there is stronger fear of COVID-19 across the board for multiple sociodemographic variables. This includes marital status, whether one is a positive case, person under investigation, or person under surveillance, and education level. However, it is not retained as a factor that results in a significant contribution to the variance for depression, anxiety, and stress, which diverges from previous findings using correlation coefficients in a similar but non-frontline population [53]. As the fear of COVID-19 is a new construct [42,54], it is interesting to see such variations appear between demographic groups. For the fear of COVID-19 between groups, positive cases have much higher levels compared to PUI and PUS groups. This may be owed to stigma, which contributes to the elevated perception of stress of being diagnosed with COVID-19 [55]; stigma has also been found to be prevalent in lay beliefs about depression in Sabahan groups [56]. Moreover, altruism and uncertainty can also contribute to our findings of higher fear of COVID-19 in healthcare frontliners [57]. Studies demonstrate healthcare workers have higher expressed fear of contracting COVID-19 [58] and adding to colleagues’ burdens [18]. Furthermore, psychological distress among hospital staff is associated with uncertainties due to frequent modifications of infection and control procedures [21]. Hence, there is an urgency to provide psychological support early on to positive cases, as they may go on to develop frank psychological sequelae later on if the fear is allowed to go unchecked. 

Furthermore, another interesting finding is that, in married people, there is significantly higher fear of COVID-19; however, at the same time, they are also more psychologically flexible and are less depressed, anxious, and stressed compared to their non-married counterparts. In light of the quarantine requirements of being a PUI or PUS, individuals who are married may have better social and logistical support in terms of grocery and food provision, and hence may have better psychological outcomes. However, they may also be more fearful of COVID-19, as they live together with other loved ones and may therefore be more fearful of causing infection. One of the risk factors for elevated emotional distress is having a family member or a colleague with confirmed COVID-19 [59,60]. Heightened psychological distress among patients with COVID-19 can also be related to fear of infecting other family members, uncertainties regarding the nature of the disease, and news media reporting the exponential rise of cases of COVID-19 and deaths of patients, which invoke fear among the patients [10,11]. 

This correlates with multiple studies that report increased prevalence of psychological distress among confirmed cases of COVID-19 patients [16,55,59,60,61,62,63]. A systematic review and meta-analysis were conducted to assess the prevalence of depression, anxiety, and sleep disturbances among patients with COVID-19, and the results revealed that the prevalence of anxiety, depression, and sleep disturbances is 47%, 45%, and 34%, respectively [64]. A study in Malaysia particularly reported that 7.5%, 7.0%, and 4.0% of the hospitalized COVID-19 patients experienced depression, anxiety, and suicidal ideation, respectively. The prevalence of depression among the hospitalized COVID-19 patients is noted to be three times higher than the national prevalence of depression (2.3%) [63].

There is no significant difference between psychological flexibility and mindfulness scores between the sociodemographic groups examined. However, for depression, anxiety, and stress, both psychological factors contribute significantly to the total variance in all three scores, whereas psychological mindedness does not have a similar contribution. This suggests that it is crucial that we perform interventions that are able to increase mindfulness and psychological flexibility, especially in frontline workers, be it in healthcare or non-healthcare settings [65]. One of the interventions that can be considered is the ultra-brief psychological intervention (UBPI). The UBPI was devised with the idea of incorporating techniques from a variety of well-established psychotherapies and enabling those useful psychological skills to be delivered to the client in a period of 15 to 20 min by the healthcare professionals. The conciseness of the module could prove to be a valuable psychological first aid instrument during these difficult times, and being a very brief intervention, it allows the healthcare professionals to utilize it with a bigger number of affected individuals. 

Interestingly, male gender proved to be a contributing factor in the hierarchical multiple regression with stress as a dependent variable, even after all psychological factors were incorporated at the second stage. Hence, there is utility in ensuring that stress in the male gender is identified, as it can be the beginning in a psychopathological pathway leading towards full-blown depressive and anxiety disorders. Evidence suggests that manifestations of such disorders in men are more insidious than in women, even though women have higher rates of depression and anxiety than men [66], and this is borne out of findings that, even though rates of suicidal behavior are higher in women, completed suicide rates are higher in men [67]. This is correlated by pandemic-age studies demonstrating that female gender and the younger population were more affected in this challenging pandemic situation [10,11,68], and further underscores the fact that a risk factor for psychological distress during the pandemic is a previous history of emotional distress [10,11].

This study has a few inherent limitations. As it is cross-sectional in design, no doubt it is only able to identify associations rather than elucidate causation pathways. Moreover, as this study assessed psychological variables using online forms, there is the possibility of response bias. The sample size is also somewhat on the low side, but this is owed to the small number of PUIs, PUSs, and positive cases in the university setting, and the potential stigma from being involved in a study examining two particularly stigmatizing states of being, that of having a diagnosable psychiatric illness, and that of having or being suspected to have COVID-19. Lastly, the study would have benefited from having randomized rather than convenience sampling techniques. However, as the numbers of PUIs and PUSs were beyond the researchers’ control, it was not possible to adequately randomize participants due to the variable number of new potential subjects in the sampling frame. There is hence room for further high impact research in future studies, chiefly in enrolling higher number of study samples across more study sites for better comparisons in a multi-site study, to assess whether different geographical locations and COVID disease burdens will have different ramifications on the psychological factors assessed in this study. 

## 5. Conclusions

In conclusion, this study again identifies the importance of the fear of COVID-19 construct, and demonstrates that there are significant differences in this construct between various sociodemographic groups, chiefly marital status and COVID-19 status. We hope that our study will be able to illustrate the magnitude of this issue and interventions can be structured and implemented accordingly to help patients cope, especially those directly affected by the COVID-19 pandemic. There is utility in primary prevention interventions, such as ultra-brief psychological interventions in the general population as part of psychological wellness, which can teach individuals without psychiatric disorders skills, such as resilience, coping with distress, appropriate ventilation of stress, and mindfulness techniques to deal with it. This will allow stress to be more normalized, and hence allow vulnerable populations to open up and reduce psychological morbidity. 

## Figures and Tables

**Table 1:** Demographic statistics of participants.

		Mean	Frequency	Percent
Age		30 years old		
Participants Status	PUI		53	29.3
	PUS		51	28.2
	POS		17	9.4
	None of above		60	33.1
Gender	Male		88	48.6
	Female		93	51.4
Educational Level	High school		40	22.1
	Diploma		28	15.5
	Bachelor		93	51.4
	Master		7	3.9
	Doctoral		13	7.2
Marital Status	Married		82	45.3
	Single		95	52.5
	Divorced		4	2.2

PUI: person under investigation. PUS: person under surveillance. POS: positive case.

**Table 2:** Bivariate correlations.

		Corrected	PM Total	CSM Total	D Score	A Score	S Score	AAQ2 Score	Total MAAS
TOTAL FCV19 corrected	Pearson Correlation	1	−0.178 *	0.436 **	0.043	0.178 *	0.099	0.088	−0.154 *
	Sig. (2-tailed)		0.017	0.000	0.568	0.017	0.186	0.241	0.038
	N	181	181	181	0.181	181	181	181	181
PM total	Pearson Correlation	−0.178 *	1	−0.238 **	−0.279 **	−0.228 **	−0.192 **	−0.255 **	0.113
	Sig. (2-tailed)	0.017		0.001	0.000	0.002	0.010	0.001	0.130
	N	181	181	181	181	181	181	181	181
CSM total	Pearson Correlation	0.436 **	−0.238 *	1	0.394 **	0.387 **	0.391 **	0.384 **	−0.238 **
	Sig. (2-tailed)	0.000	0.001		0.000	0.000	0.000	0.000	0.001
	N	181	181	181	181	181	181	181	181
D score	Pearson Correlation	0.043	−0.279 **	0.394 **	1	0.753 **	0.774 **	0.687 **	−0.449 **
	Sig. (2-tailed)	0.568	0.000	0.000		0.000	0.000	0.000	0.000
	N	181	181	181	181	181	181	181	181
A score	Pearson Correlation	0.178 *	−0.228 **	0.387 **	0.753 **	1	0.843 **	0.650 **	−0.505 **
	Sig. (2-tailed)	0.017	0.002	0.000	0.000		0.000	0.000	0.000
	N	181	181	181	181	181	181	181	181
S score	Pearson Correlation	0.099	−0.192 **	0.391 **	0.774 **	0.843 **	1	0.729 **	−0.502 **
	Sig. (2-tailed)	0.186	0.010	0.000	0.000	0.000		0.000	0.000
	N	181	181	181	181	181	181	181	181
AAQ2 score	Pearson Correlation	0.088	−0.255 **	0.384 **	0.687 **	0.650 **	0.729 **	1	−0.459 **
	Sig. (2-tailed)	0.241	0.001	0.000	0.000	0.000	0.000		0.000
	N	181	181	181	181	181	181	181	181
Total MAAS	Pearson Correlation	−0.154 *	0.113	−0.238 **	−0.449 **	−0.505 **	−0.502 **	−0.459 **	1
	Sig. (2-tailed)	0.038	0.130	0.001	0.000	0.000	0.000	0.000	
	N	181	181	181	181	181	181	181	181

* Correlation is significant at the 0.05 level (two-tailed); ** Correlation is significant at the 0.001 level (two-tailed).

**Table 3:** Ranks based on surveillance status.

	Status	N	Mean Rank
TOTAL FCV19 corrected	PUI	53	75.87
	PUS	51	78.18
	POS	17	119.29
	none of above	60	96.44
	Total	181	
PM total	PUI	53	75.61
	PUS	51	91.85
	POS	17	89.82
	none of above	60	92.92
	Total	181	
Total CSM	PUI	53	81.45
	PUS	51	94.90
	POS	17	75.38
	None of above	60	88.70
	Total	181	
Total MAAS	PUI	53	90.11
	PUS	51	89.07
	POS	17	95.06
	none of above	60	79.16
	Total	181	
AAQ2 score	PUI	53	81.69
	PUS	51	94.35
	POS	17	72.12
	none of above	60	90.07
	Total	181	
D score	PUI	53	91.55
	PUS	51	87.55
	POS	17	66.97
	none of above	60	88.38
	Total	181	
A score	PUI	53	86.77
	PUS	51	89.31
	POS	17	79.15
	none of above	60	87.53
	Total	181	
S score	PUI	53	91.42
	PUS	51	87.83
	POS	17	68.56
	none of above	60	87.71
	Total	181	

**Table 4:** Test statistics based on Table 3.

	Total FCV19 Corrected	PM Total	Total CSM	Total MAAS	AAQ2 Score	D Score	A Score	S Score
Kruskal–Wallis H	13.141	4.011	2.911	2.007	3.396	3.327	0.542	2.759
df	3	3	3	3	3	3	3	3
Asymp. Sig.	0.004	0.260	0.406	0.571	0.334	0.344	0.910	0.430

**Table 5:** Ranks based on marital status.

	Marital Status	N	Mean Rank
TOTAL FCV19 corrected	Married	82	105.91
	Single	95	78.11
	Divorced	4	91.50
	Total	181	
PM total	Married	82	92.70
	Single	95	91.23
	Divorced	4	50.63
	Total	181	
Total CSM	Married	82	91.59
	Single	95	91.12
	Divorced	4	76.13
	Total	181	
Total MAAS	Married	82	98.80
	Single	95	83.64
	Divorced	4	105.88
	Total	181	
AAQ2 score	Married	82	70.01
	Single	95	108.78
	Divorced	4	99.00
	Total	181	
D score	Married	82	75.86
	Single	95	104.22
	Divorced	4	87.38
	Total	181	
A score	Married	82	78.30
	Single	95	102.95
	Divorced	4	67.50
	Total	181	
S score	Married	82	78.11
	Single	95	102.71
	Divorced	4	77.13
	Total	181	

**Table 6:** Test statistics based on Table 5.

	Total FCV19 Corrected	PM Total	Total CSM	Total MAAS	AAQ2 Score	D Score	A Score	S Score
Kruskal–Wallis H	12.411	2.471	0.335	4.022	24.240	13.410	10.742	10.055
df	2	2	2	2	2	2	2	2
Asymp. Sig.	0.002	0.291	0.846	0.134	0.000	0.001	0.005	0.007

**Table 7:** Ranks based on education levels.

	Educational Level	N	Mean Rank
TOTAL FCV19 corrected	High school	40	126.29
	Diploma	28	117.91
	Bachelor	93	76.05
	Master	7	39.71
	Doctoral	13	59.04
	Total	181	
PM total	High school	40	93.14
	Diploma	28	65.27
	Bachelor	93	98.31
	Master	7	80.71
	Doctoral	13	93.12
	Total	181	
Total CSM	High school	40	90.10
	Diploma	28	104.46
	Bachelor	93	87.92
	Master	7	64.14
	Doctoral	13	101.23
	Total	181	
Total MAAS	High school	40	107.58
	Diploma	28	90.20
	Bachelor	93	82.45
	Master	7	102.29
	Doctoral	13	96.85
	Total	181	
AAQ2 score	High school	40	76.61
	Diploma	28	83.13
	Bachelor	93	102.90
	Master	7	48.57
	Doctoral	13	89.96
	Total	181	
D score	High school	40	77.51
	Diploma	28	91.98
	Bachelor	93	101.09
	Master	7	58.29
	Doctoral	13	75.85
	Total	181	
A score	High school	40	81.96
	Diploma	28	84.98
	Bachelor	93	100.08
	Master	7	62.71
	Doctoral	13	82.08
	Total	181	
S score	High school	40	76.45
	Diploma	28	91.29
	Bachelor	93	101.46
	Master	7	69.57
	Doctoral	13	71.85
	Total	181	

**Table 8:** Test statistics based on Table 7.

	Total FCV19 Corrected	PM Total	Total CSM	Total MAAS	AAQ2 Score	D Score	A Score	S Score
Kruskal–Wallis H	44.729	8.945	4.543	6.981	13.060	10.303	6.879	9.765
df	4	4	4	4	4	4	4	4
Asymp. Sig.	0.000	0.062	0.337	0.137	0.011	0.036	0.142	0.045

**Table 9:** Hypothesis test summary.

	Null Hypothesis	Test	Sig.	Decision
1	The distribution of CSM total is the same across categories of gender.	IndependentSamples Mann–Whitney U Test	0.002	Reject the null hypothesis.
2	The distribution of total FCV19 corrected is the same across categories of gender.	IndependentSamples Mann–Whitney U Test	0.596	Retain the null hypothesis.
3	The distribution of PM total is the same across categories of gender.	IndependentSamples Mann–Whitney U Test	0.059	Retain the null hypothesis.
4	The distribution of Total MAAS is the same across categories of gender.	IndependentSamples Mann–Whitney U Test	0.164	Retain the null hypothesis.
5	The distribution of total AAQ2 is the same across categories of gender.	IndependentSamples Mann–Whitney U Test	0.028	Reject the null hypothesis.
6	The distribution of the D score is the same across categories of gender.	IndependentSamples Mann–Whitney U Test	0.011	Reject the null hypothesis.
7	The distribution of the A score is the same across categories of gender.	IndependentSamples Mann–Whitney U Test	0.005	Reject the null hypothesis.
8	The distribution of the S score is the same across categories of gender.	IndependentSamples Mann–Whitney U Test	0.001	Reject the null hypothesis.

**Table 10:** Model summary for depression as the dependent variable.

Model	R	R Square	Adjusted R Square	Std. Error of the Estimate	Change Statistics				
					R Square Change	F Change	df1	df2	Sig. F Change
1	0.356 ^a^	0.127	0.067	4.274	0.127	2.130	11	161	0.021
2	0.745 ^b^	0.555	0.509	3.102	0.428	29.945	5	156	0.000

^a^ Predictors: (Constant), POS, Diploma, Divorced, Master, Doctoral, Male, PUI, Married, PUS, Bachelor, Age. ^b^ Predictors: (Constant), POS, Diploma, Divorced, Master, Doctoral, Male, PUI, Married, PUS, Bachelor, Age, Total MAAS, PM total, CSM total, AAQ2 score, TOTAL FCV19 corrected.

**Table 11:** ANOVA ^a^ based on Table 10.

Model		Sum of Squares	df	Mean Square	F	Sig.
1	Regression	428.106	11	38.919	2.130	0.021 ^b^
	Residual	2941.663	161	18.271		
	Total	3369.769	172			
2	Regression	1868.752	16	116.797	12.139	0.000 ^c^
	Residual	1501.017	156	9.622		
	Total	3369.769	172			

^a^ Dependent Variable: D score. ^b^ Predictors: (Constant), POS, Diploma, Divorced, Master, Doctoral, Male, PUI, Married, PUS, Bachelor, Age. ^c^ Predictors: (Constant), POS, Diploma, Divorced, Master, Doctoral, Male, PUI, Married, PUS, Bachelor, Age, Total MAAS, PM total, CSM total, AAQ2 score, TOTAL FCV19 corrected.

**Table 12:** Model summary for anxiety as dependent variable.

Model	R	R Square	Adjusted R Square	Std. Error of the Estimate	Change Statistics				
					R Square Change	F Change	df1	df2	Sig. F Change
1	0.343 ^a^	0.118	0.058	4.296	0.118	1.955	11	161	0.036
2	0.716 ^b^	0.513	0.463	3.242	0.395	25.331	5	156	0.000

^a^ Predictors: (Constant), POS, Diploma, Divorced, Master, Doctoral, Male, PUI, Married, PUS, Bachelor, Age. ^b^ Predictors: (Constant), POS, Diploma, Divorced, Master, Doctoral, Male, PUI, Married, PUS, Bachelor, Age, Total MAAS, PM total, CSM total, AAQ2 score, TOTAL FCV19 corrected.

**Table 13:** ANOVA ^a^ based on Table 12.

Model		Sum of Squares	df	Mean Square	F	Sig.
1	Regression	396.939	11	36.085	1.955	0.036 ^b^
	Residual	2971.027	161	18.454		
	Total	3367.965	172			
2	Regression	1728.231	16	108.014	10.276	0.000 ^c^
	Residual	1639.734	156	10.511		
	Total	3367.965	172			

^a^ Dependent Variable: A score. ^b^ Predictors: (Constant), POS, Diploma, Divorced, Master, Doctoral, Male, PUI, Married, PUS, Bachelor, Age. ^c^ Predictors: (Constant), POS, Diploma, Divorced, Master, Doctoral, Male, PUI, Married, PUS, Bachelor, Age, Total MAAS, PM total, CSM total, AAQ2 score, TOTAL FCV19 corrected.

**Table 14:** Coefficients ^a^.

Model		Unstandardized Coefficients		Standardized Coefficients	t	Sig.
		B	Std. Error	Beta		
1	(Constant)	9.352	1.928		4.851	0.000
	Age	−0.127	0.058	−0.232	−2.207	0.029
	Male	−1.457	0.706	−0.165	−2.063	0.041
	Married	−0.542	0.933	−0.061	−0.581	0.562
	Divorced	0.521	2.642	0.015	0.197	0.844
	Diploma	−0.458	1.140	−0.038	−0.402	0.688
	Bachelor	−0.023	0.909	−0.003	−0.026	0.979
	Master	−1.033	2.116	−0.039	−0.488	0.626
	Doctoral	−0.185	1.447	−0.011	−0.128	0.898
	PUI	−0.244	0.873	−0.026	−0.280	0.780
	PUS	−0.250	0.917	−0.026	−0.273	0.785
	POS	−0.269	1.239	−0.018	−0.217	0.829
2	(Constant)	4.340	1.868		1.513	0.132
	Age	0.017	0.046	0.031	0.375	0.708
	Male	−0.753	0.562	−0.085	−1.339	0.183
	Married	−0.556	0.719	−0.063	−0.772	0.441
	Divorced	−1.161	2.042	−0.034	−0.568	0.571
	Diploma	−0.618	0.870	−0.051	−0.710	0.478
	Bachelor	−0.011	0.746	−0.001	−0.015	0.988
	Master	−0.174	1.672	−0.007	−0.104	0.917
	Doctoral	−0.500	1.162	−0.030	−0.430	0.668
	PUI	0.876	0.683	0.092	1.283	0.201
	PUS	0.297	0.705	0.031	0.421	0.675
	POS	1.123	0.967	0.076	1.161	0.247
	CSM total	0.100	0.071	0.105	1.405	0.162
	TOTAL FCV19 corrected	0.018	0.027	0.053	0.672	0.503
	PM total	−0.019	0.045	−0.027	−0.430	0.668
	Total MAAS	−0.081	0.020	−0.258	−4.001	0.000
	AAQ2 score	0.213	0.035	0.468	6.035	0.000

^a^ Dependent Variable: A score.

**Table 15:** Model summary for stress as dependent variable.

Model	R	R Square	Adjusted R Square	Std. Error of the Estimate	Change Statistics				
					R Square Change	F Change	df1	df2	Sig. F Change
1	0.396 ^a^	0.157	0.100	4.566	0.157	2.728	11	161	0.003
2	0.779 ^b^	0.607	0.567	3.168	0.450	35.698	5	156	0.000

^a^ Predictors: (Constant), POS, Diploma, Divorced, Master, Doctoral, Male, PUI, Married, PUS, Bachelor, Age. ^b^ Predictors: (Constant), POS, Diploma, Divorced, Master, Doctoral, Male, PUI, Married, PUS, Bachelor, Age, Total MAAS, PM total, CSM total, AAQ2 score, TOTAL FCV19 corrected.

**Table 16:** ANOVA ^a^ based on Table 15.

Model		Sum of Squares	df	Mean Square	F	Sig.
1	Regression	625.609	11	56.874	2.728	0.003 ^b^
	Residual	3356.310	161	20.847		
	Total	3981.919	172			
2	Regression	2416.592	16	151.037	15.052	0.000 ^c^
	Residual	1565.327	156	10.034		
	Total	3981.919	172			

^a^ Dependent Variable: S score. ^b^ Predictors: (Constant), POS, Diploma, Divorced, Master, Doctoral, Male, PUI, Married, PUS, Bachelor, Age. ^c^ Predictors: (Constant), POS, Diploma, Divorced, Master, Doctoral, Male, PUI, Married, PUS, Bachelor, Age, Total MAAS, PM total, CSM total, AAQ2 score, TOTAL FCV19 corrected.

**Table 17:** Coefficients ^a^.

Model		Unstandardized Coefficients		Standardized Coefficients	t	Sig.
		B	Std. Error	Beta		
1	(Constant)	11.834	2.049		5.776	0.000
	Age	−0.176	0.061	−0.295	−2.872	0.005
	Male	−1.926	0.751	−0.201	−2.567	0.011
	Married	0.003	0.991	0.000	0.003	0.997
	Divorced	0.972	2.808	0.026	0.346	0.730
	Diploma	0.331	1.211	0.025	0.273	0.785
	Bachelor	0.489	0.966	0.051	0.506	0.614
	Master	0.551	2.249	0.019	0.245	0.807
	Doctoral	−0.268	1.538	−0.015	−0.174	0.862
	PUI	−0.137	0.928	−0.013	−0.148	0.882
	PUS	0.122	0.975	0.012	0.125	0.901
	POS	−1.535	1.316	−0.095	−1.166	0.245
2	(Constant)	2.309	2.803		0.824	0.411
	Age	0.008	0.045	0.014	0.188	0.851
	Male	−1.169	0.549	−0.122	−2.127	0.035
	Married	0.220	0.703	0.023	0.313	0.755
	Divorced	−0.891	1.995	−0.024	−0.447	0.656
	Diploma	0.243	0.850	0.018	0.286	0.775
	Bachelor	0.173	0.728	0.018	0.237	0.813
	Master	1.348	1.634	0.047	0.825	0.411
	Doctoral	−1.068	1.135	−0.059	−0.941	0.348
	PUI	1.276	0.667	0.123	1.914	0.057
	PUS	0.766	0.689	0.073	1.112	0.268
	POS	0.319	0.945	0.020	0.338	0.736
	CSM total	0.094	0.070	0.090	1.348	0.180
	TOTAL FCV19 corrected	−0.004	0.026	−0.009	−0.134	0.894
	PM total	0.037	0.044	0.048	0.848	0.398
	Total MAAS	−0.070	0.020	−0.205	−3.535	0.001
	AAQ2 score	0.308	0.034	0.623	8.936	0.000

^a^ Dependent Variable: S score.

## Data Availability

The data presented in this study are available on request from the corresponding author. The data are not publicly available due to privacy issues.
